# Metal(loid)s and their bioaccessibility in urban soils from residential areas of a medieval mining town

**DOI:** 10.1007/s10653-024-02339-y

**Published:** 2025-02-01

**Authors:** Vojtěch Ettler, Jitka Waldhauserová, Petr Drahota, Marek Tuhý, Martin Mihaljevič, Martin Racek

**Affiliations:** 1 Institute of Geochemistry, Mineralogy and Mineral Resources, Faculty of Science, Charles University, Albertov 6, Prague, 128 00 Czech Republic; 2 Commonwealth Scientific and Industrial Research Organization (CSIRO), GPO Box 1700, Canberra,ACT, Australia; 3 Institute of Petrology and Structure Geology, Faculty of Science, Charles University, Albertov 6, Prague, 128 00 Czech Republic

**Keywords:** Contamination, Exposure, Human health, Oral ingestion

## Abstract

In historic mining towns, where mining activities were abandoned many decades or even centuries ago, legacy contaminations can be remobilized and redispersed, representing a threat for the environment and human health. This study focuses on urban soils (*n* = 19) in the town of Jihlava, the Czech Republic, one of the medieval centers of silver mining in central Europe. The basic geochemical characterization of the soils was combined with mineralogical investigations to understand the solid speciation of the metal(loid) contaminants, oral bioaccessibility tests, and exposure assessment. The total concentrations of the metal(loid)s in the original soils were not excessively high (up to 45.8 mg As/kg, 19.2 mg Cd/kg; 205 mg Cr/kg; 91.8 mg Cu/kg, 163 mg Pb/kg, 253 mg V/kg, 262 mg Zn/kg), although, in some cases, they exceeded the regulatory guidelines for agricultural and/or residential soils. A substantial increase in the metal(loid)s contents was confirmed for the < 48-µm soil fraction that was later used for the bioaccessibility tests. Scanning electron microscopy and the electron microprobe showed that ore-derived primary sulfides were rare in the studied soils. Still, hydrous ferric oxides rich in Cu, Pb and Zn and fragments of metallurgical slags composed of metal-containing glass and silicates (olivine) were prone to dissolution during extraction in a simulated gastric fluid (SGF, glycine solution acidified to pH 1.5 by HCl). The maximum bioaccessible concentrations corresponded to 4.69 mg As/kg, 1.75 mg Cd/kg, 2.02 mg Cr/kg, 20.3 mg Cu/kg, 81.6 mg Pb/kg, 16.2 mg V/kg, and 233 mg Zn/kg. Exposure estimates were carried out for children (10 kg) as a target group and a conservative soil ingestion rate (100 mg/d). However, the daily intake of all the studied contaminants was far below the tolerable limits. Our results show that the human health risk based on incidental soil ingestion in the studied area seems limited.

**Supplementary Information:**

The online version contains supplementary material available at 10.1007/s10653-024-02339-y.

## Introduction

Contamination in urban areas affected by heavy traffic and various industrial activities has been studied in numerous places on Earth and multielement geochemistry and isotopic approaches have often been used for the source apportionment of potentially toxic metals and metalloids in dust and soils (see, e.g., Galušková et al., 2014; Hiller et al., 2017; Kelepertzis et al., 2021; Tyszka et al., 2024). Human health issues related to contaminated dust and soil dispersion are particularly critical in urban areas (Gbefa et al., 2011; Juhasz et al., 2011; Kelepertzis et al., 2021; Madrid et al., 2008a, 2008b; Okorie et al., 2012; Reis et al., 2014a, 2014b, 2018).

Many urban settlements have developed near mining operations, where local populations are directly exposed to metal(loid)s bound in dust particles, either emitted by the ore mining and processing activities themselves or resuspended from the contaminated soils. Active mining and smelting areas have especially been identified as sources of metalliferous dust, which can be potentially inhaled, ingested, or adhere to crop surfaces (Argyraki, 2014; Fry et al., 2020; Goix et al., 2016; Hu et al., 2019; Soto-Jiménez et al., 2023). For example, a study from the Zambian Copperbelt compared the metal(loid) bioaccessibility from topsoils in mining and smelting areas and demonstrated that a more severe risk exposure is expected in smelter-affected sites, where contaminants are more soluble in simulated gastric fluid (Ettler et al., 2012). Health risks can persist even in the vicinity of abandoned/closed mining operations due to legacy contamination related to waste disposal sites (mine tailings, slags, residues) and polluted soils (Boisa et al., 2013; Harvey et al., 2016; Roussel et al., 2010).

However, the question arises: what happens in places where mining activities were abandoned many decades or even centuries ago? There are still existing geochemical anomalies in historical mining districts, and many urban activities, such as the construction industry, can remobilize and redisperse the legacy contaminations (e.g., buried old mine and metallurgical wastes, polluted soils). Exposure assessments in historical mining districts, where ore mining activities peaked a long time ago (Middle Ages), are still quite rare in the literature (Drahota et al., 2018). To fill this gap, this paper focuses on urban soils from the town of Jihlava, one of the medieval centers of silver mining of the Kingdom of Bohemia (the original name of the territory corresponding to the current Czech Republic). This town has been studied from the archeological point of view (e.g., Hrubý, 2019), but environmental studies focusing on soils in the most frequented areas of the agglomeration center have not been carried out yet. The objectives of this study are (i) to determine the bulk concentrations, solid speciation, and oral bioaccessibilities of the metal(loid)s in the topsoils from the residential environment collected near schools, kindergartens, sports areas, and frequented parks and (ii) to assess the risk related to the potential ingestion of fine soil fractions considering children as the main targets.

## Materials and methods

### Study area

**Fig. 1:**
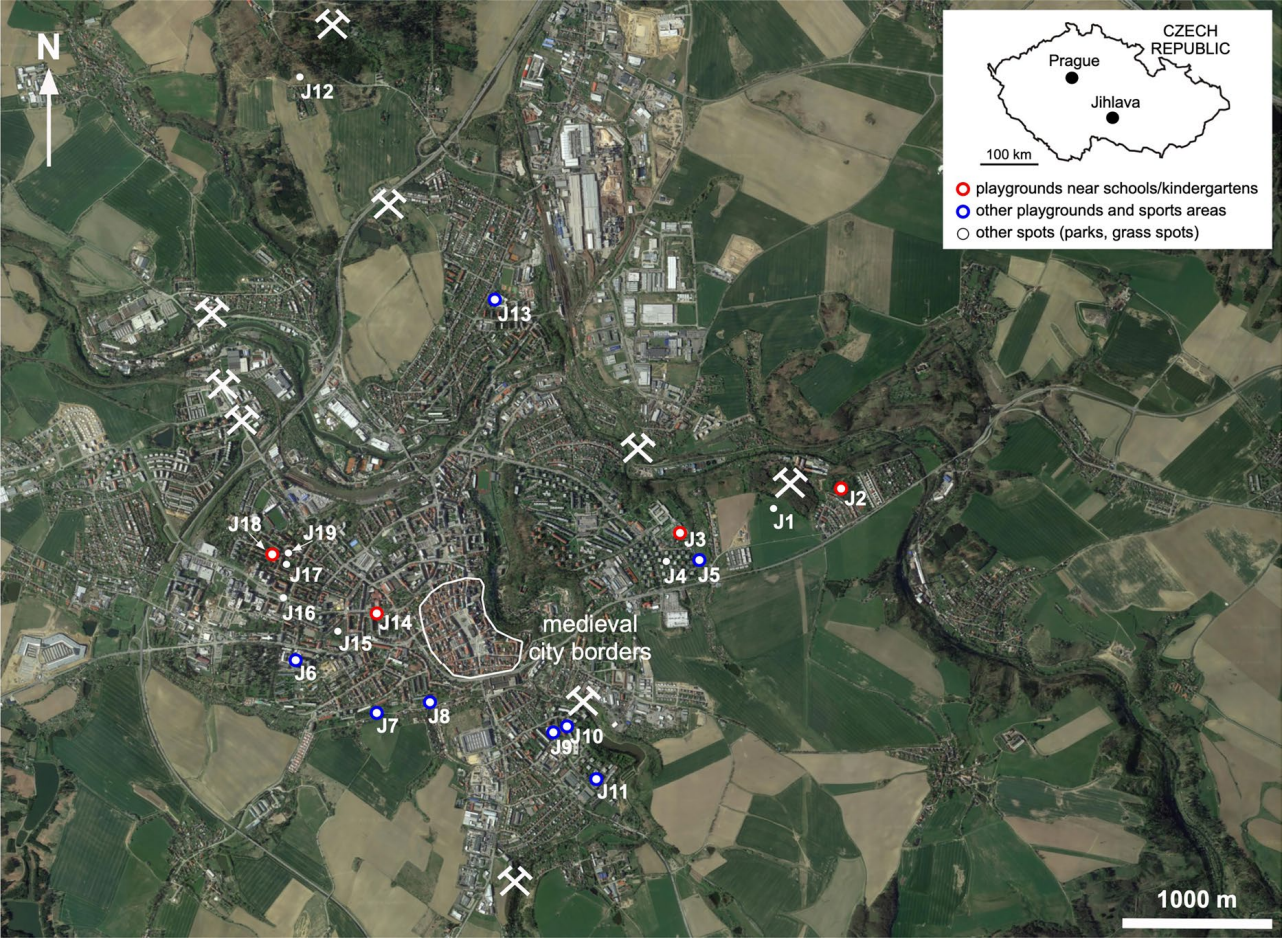
Map of the studied area (based on Google Earth™) with an indication of the soil sampling sites, the medieval borders of the Jihlava town, and former mining areas (the latter information is based on Koutek, (1952) and Hrubý, (2019). For the GPS coordinates and detailed characteristics of the soil sampling points, see the Supplementary Information (Table S1)

The town of Jihlava, located approximately 115 km SE of Prague, the capital of the Czech Republic, belonged to one of the important medieval silver mining centers in the Bohemian-Moravian Highlands. The ore veins of variable sizes (a few centimeters to two meters) composed mainly of sulfides (Ag-bearing galena, PbS, sphalerite, ZnS, pyrite, FeS_2_ and less abundant chalcopyrite, CuFeS_2_, and arsenopyrite, FeAsS) with predominant quartz filling penetrated the gneiss rocks in the N-S direction (Koutek, 1952; Vosáhlo, 1988). The Jihlava historic mining district was one of Europe’s most important silver producing areas, mainly in the thirteenth and fourteenth centuries (Holub, 2015). It was probably established before 1250 AD, and the oldest reference to mining activities in Staré Hory, located ca 2 km NW of the town center, dates to 1315 AD (Derner et al., 2019; Hrubý, 2019). Archeological excavations discovered many mining centers around the old Jihlava town (see also Fig. 1) as well as metallurgical remains (furnace remains, slags, ingots, and drop-offs) dated to this period (see, e.g., Kapusta et al., 2017, 2022; Hrubý, 2019).

The second and third phases of silver mining in the studied area correspond to the periods 1436–1618 and the 1660s–1780s, respectively. The last large-scale prospecting works were carried out in 1769–1783 (Koutek, 1952; Pluskal & Vosáhlo, 1998). The overall decline of the silver mining activities in the eighteenth century in the Jihlava district was affected mainly by the extensive import of large amounts of Ag from South American mining sites.

### Soil sampling and processing

In this study, we focused on samples from the wider agglomeration of Jihlava (Fig. 1) to assess the effects of the former mining activities on the environmental quality of urban soils with a focus on the potential bioaccessibility of metal(loid)s. A total of 19 soil samples were collected (Fig. 1, Table S1) with an emphasis on places where the local population (especially children) can be in contact with the soil. For this purpose, we collected soils near schools, kindergartens, sports areas, and frequented parks, not necessarily in the close vicinity of the former shafts and mining/metallurgical remains, which, in many cases, remain in the industrial areas or are hidden in the forest outside the main agglomeration (Fig. 1, Table S1). For soil sampling, we used the EuroGeoSurveys methodology based on a 10 × 10 m grid to collect five subsamples (in each corner and in the center of this square) to obtain one composite sample (GEMAS, 2008). Approximately 1–2 kg of the topsoil (depth: 0–15 cm) was collected at each spot using an auger drill (Eijkelkamp, the Netherlands).

In the laboratory, the samples were air-dried and sieved to < 2 mm (Retsch stainless steel sieve, Germany). Traditionally, a < 250 µm soil fraction has been used for mimicking the hand-to-mouth incidental soil ingestion. Still, the United States Environmental Protection Agency (US EPA) has recently lowered the particle size fraction to < 150 µm for bioaccessibility testing (US EPA, 2017). Moreover, many experimental studies have demonstrated that even smaller particles adhere to surfaces on hands and recommend that fractions ~ 45 µm should be used (for rationale, see, e.g., Siciliano et al., 2009; Yamamoto et al., 2006; Li et al., 2021; Ettler et al., 2022). For this reason, the soil fraction < 48 µm used subsequently for the bioaccessibility testing was obtained by dry sieving (48-µm polyamide sieve UHELON 120 T, producer: Silk & Progress, Brněnec, the Czech Republic). An aliquot of each soil sample (< 2 mm and < 48 µm), for determining the total elemental concentrations, was ground to a fine powder in an agate mortar (Retsch planetary mill PM 400, Germany).

### Geochemical and mineralogical characterization

The total concentrations of metal(loid)s in the soils (both granulometric fractions) were measured by a portable X-ray fluorescence spectrometer (pXRF; Thermo Scientific Niton XL3t Goldd instrument with a Thermo Scientific sample holder, calibration mode: AllGeo, acquisition time: 4 × 40 s). The bulk chemical analysis was focused on As, Cd, Cr, Cu, Fe, Mn, Pb, V, and Zn. The quality control/quality assurance (QC/QA) procedure of the bulk chemistry determinations using pXRF was controlled by parallel analyses of multiple standard reference materials (soils SRM 2709a, 2710a, 2711a certified by the National Institute of Standards and Technology [NIST], USA) and was found to be satisfactory (Table S2).

For the detailed mineralogical investigations, the heavy mineral fractions of the selected original soils [samples J1, J12, J14, and J15 exhibiting the highest total concentrations of metal(loid)s] were obtained using 1,1,2,2-tetrabromoethane (density 2.96 g/cm^3^) after centrifuging at 1800 rpm (Janetzki S70D, Germany) (Tuhý et al., 2020). These heavy mineral fractions of soils were embedded in epoxy resin, prepared as polished sections, and examined using an optical microscope (Leica MPS60 DM LP, Germany, and Zeiss Axio Imager.A2m with Axiocam 305 camera, Germany). For the elemental distribution in the polished sections, we used micro-X-ray fluorescence spectrometry (μ-XRF; Bruker M4 TORNADO, Germany, equipped with a Rh X-ray source operating at 50 kV and 400 mA). The cumulative energy dispersion spectra obtained by μ-XRF were recorded in two cycles with a step of 20 μm and an acquisition time of 100 ms (the measurement of one polished section was completed in approximately 8 h). The polished sections were further examined by an electron probe microanalyzer (EPMA; JEOL JXA-8530F, Japan) equipped with a field emission gun electron source (FEG). This instrument was used for the scanning electron microscopy (SEM) images, energy dispersion spectroscopy (EDS) analyses (JEOL JED-2300F spectrometer), and quantitative chemical analyses of the individual minerals using wavelength dispersion spectroscopy (WDS). The EPMA analytical conditions, standards, and detection limits are given in Table S2.

### Bioaccessibility testing and exposure assessment

The oral bioaccessibility test, corresponding to a gastric phase extraction and representing a “worst-case scenario” in the soil exposure assessment, was performed according to the US Environmental Protection Agency (EPA)’s experimental protocol (2017). The < 48-µm soil fraction was extracted in a simulated gastric fluid (SGF) composed of a 0.4 M glycine solution adjusted to pH 1.5 ± 0.05 by HCl (reagent grade, Merck, Germany) at a liquid-to-solid (L/S) ratio of 100 (0.1 g of soil to 10 ml of extracting solution). The mixtures were agitated for 1 h at 37 °C in a GFL 3032 incubator (GFL, Germany). The extractions were performed in duplicate and with procedural blanks. The extracts were filtered through a 0.45-µm membrane filter (using Millipore Millex-HV PVDF Durapore disposable filters, USA and B-Braun Omnifix 20 ml luer-lock syringes, Germany). The solutions obtained were then diluted in 2% HNO_3_ (v/v) and analyzed for the selected metal(loid)s (As, Cd, Cr, Cu, Fe, Mn, Pb, V, Zn) by the quadrupole-based inductively coupled plasma mass spectrometry (ICP-MS; ThermoScientific, iCAP-Q™, Germany). Finally, the bioaccessible concentrations of the metal(loid)s were expressed in mg/kg and converted to a bioaccessible fraction (BAF; percentage of the total contents).

The standard reference material NIST SRM 1463f (Trace elements in water) was used to verify the accuracy of the measurements by ICP-MS (Table S2). Furthermore, to verify the accuracy of the bioaccessible extraction, NIST SRM 2710a and 2711a were extracted using SGF, and the bioaccessible concentrations of Pb and As were compared with the certified and published values (Dodd et al., 2024; US EPA, 2017 (Table S2).

The exposure calculations were based on children (weighing 10 kg) being considered as a potential target group in the urban area studied. The soil and dust ingestion rates used for the exposure estimates found in the literature are rather variable; for example, Özkaynak et al. (2011) reported that for children 3 to < 6 years of age, the mean and the 95th percentile of total soil ingestion correspond to 68 and 224 mg/d, respectively. Based on a detailed statistical evaluation, they calculated that the mean soil ingestion is 41 mg/d. However, the conservative daily soil/dust intake used as a model in this type of exposure assessment generally corresponds to 100 mg/d for children (Bierkens et al., 2011), and we also adopted it in this study. However, we have also evaluated an exposure scenario with soil/dust intake of 1000 mg/d, proposed for soil ‘pica’ behavior (Moya & Phillips, 2014). Minimal risk levels (MRLs) defined by the US Agency for Toxic Substances and Disease Registry (ATSDR, 2024) were used for the exposure assessment (µg/kg_bw_/d, exposure duration) (bw = body weight): As (5, acute), Cd (0.5, acute), Cr^VI^ (5, acute), Cu (20, acute; recently increased from 10 to 20), V (10, intermediate), and Zn (300, intermediate). Furthermore, the obtained daily intakes of metal(oid)s were also compared with the tolerable daily intake (TDI) limits taken from Baars et al. (2001) and Tiesjema and Baars (2009) (µg/kg_bw_/d): As (1), Cd (0.5), Cr^VI^ (5), Cu (140), Pb (3.6), V (2), and Zn (500). This has been undertaken even though the European Food Safety Authority (EFSA) has lowered the TDI for Cd to 0.36 µg/kg_bw_ (EFSA, 2009) and suggested that the provisional TDI limit for Pb (3.6 µg/kg_bw_) is no longer appropriate (EFSA, 2010).

### Data processing

The data were plotted using a combination of the Prism 10 (GraphPad, USA) and Graphic for Mac (Picta, USA) software packages. Prism 10 was also used for the statistical data treatment. The normality of data was assessed using the Shapiro–Wilk test (alpha = 0.05) followed either by the paired t-test (for the data with normal distribution) or Wilcoxon matched-pairs signed rank test (for the data with lognormal distribution) (alpha = 0.05). The geochemical code PHREEQC-3 for macOS (Parkhurst & Appelo, 2013) was used for the speciation-solubility modeling to calculate the degree of saturation of the bioaccessibility extracts with respect to the potential solubility-controlling phases (i.e., the calculation of the saturation index, SI). The minteq.v4.dat thermodynamic database was used for all the calculations (pH was set at 1.5 and pe at 12, corresponding to an Eh value of approximately 700 mV, which is common in SGF extracts; see Ettler et al., 2020).

## Results and discussion

### Bulk concentrations

The concentrations of trace elements in the original soil samples varied as follows (mg/kg): As: < 7–45.8, Cd: < 12–19.2; Cr: 84–205; Cu < 13–91.8; Pb: 21.7–163; V: < 25–253; Zn: 25.7–262. Surprisingly, these values were not excessively high compared to soils from other former mining/smelting towns (e.g., Drahota et al., 2018; Harvey et al., 2016; Roussel et al., 2010), but, in some cases, exceeded the Canadian Soil Quality Guidelines for residential/parkland soils for As (12 mg/kg, 6 samples), Cd (10 mg/kg, 3 samples above the LOD of 12 mg/kg), Cr (64 mg/kg, all the samples), Pb (140 mg/kg, 1 sample) and V (130 mg/kg, 9 samples) (CCME, 2024; see Table S4 in the Supplementary Material).

**Fig. 2:**
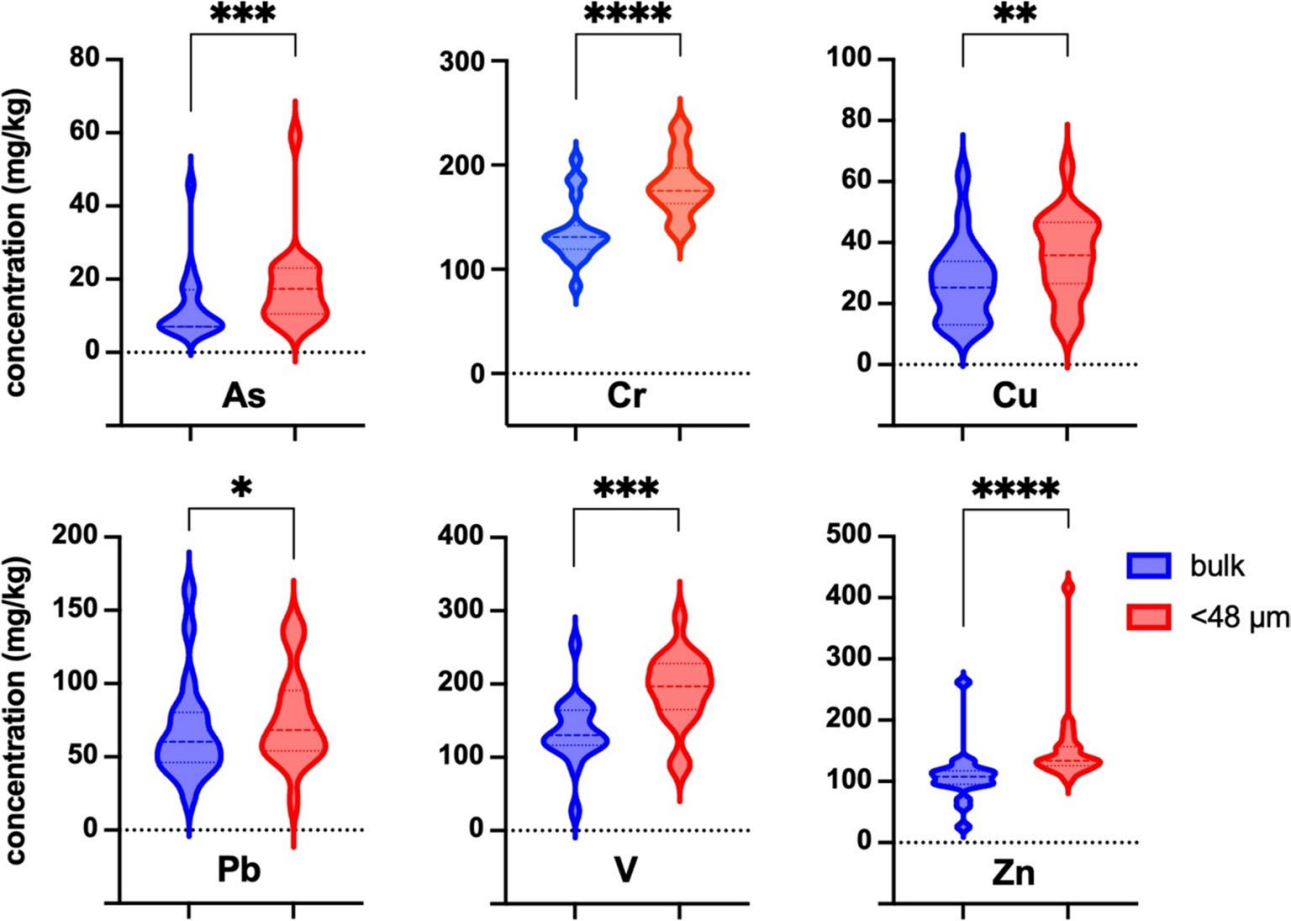
Violin plots indicate the differences between the metal(loid) concentrations in the bulk soils and the < 48 µm soil fractions. Calculations using the Wilcoxon matched-pairs signed rank test (As, Cr, Pb, Zn) or the paired t-test (Cu, V) (P < 0.05) indicate that the differences are statistically significant for all the elements. Asterisks correspond to the P-values classification: * ≤ 0.05; ** ≤ 0.01; *** ≤ 0.001; **** ≤ 0.0001

The trace elements concentration was higher for the < 48-µm soil fractions compared to the bulk soils, and, for the key contaminants, these differences were statistically significant (Fig. 2). A similar phenomenon was also observed in many other studies (see, e.g., Madrid et al., 2008a; Ettler et al., 2019).

### Binding of metals and metalloids

**Fig. 3:**
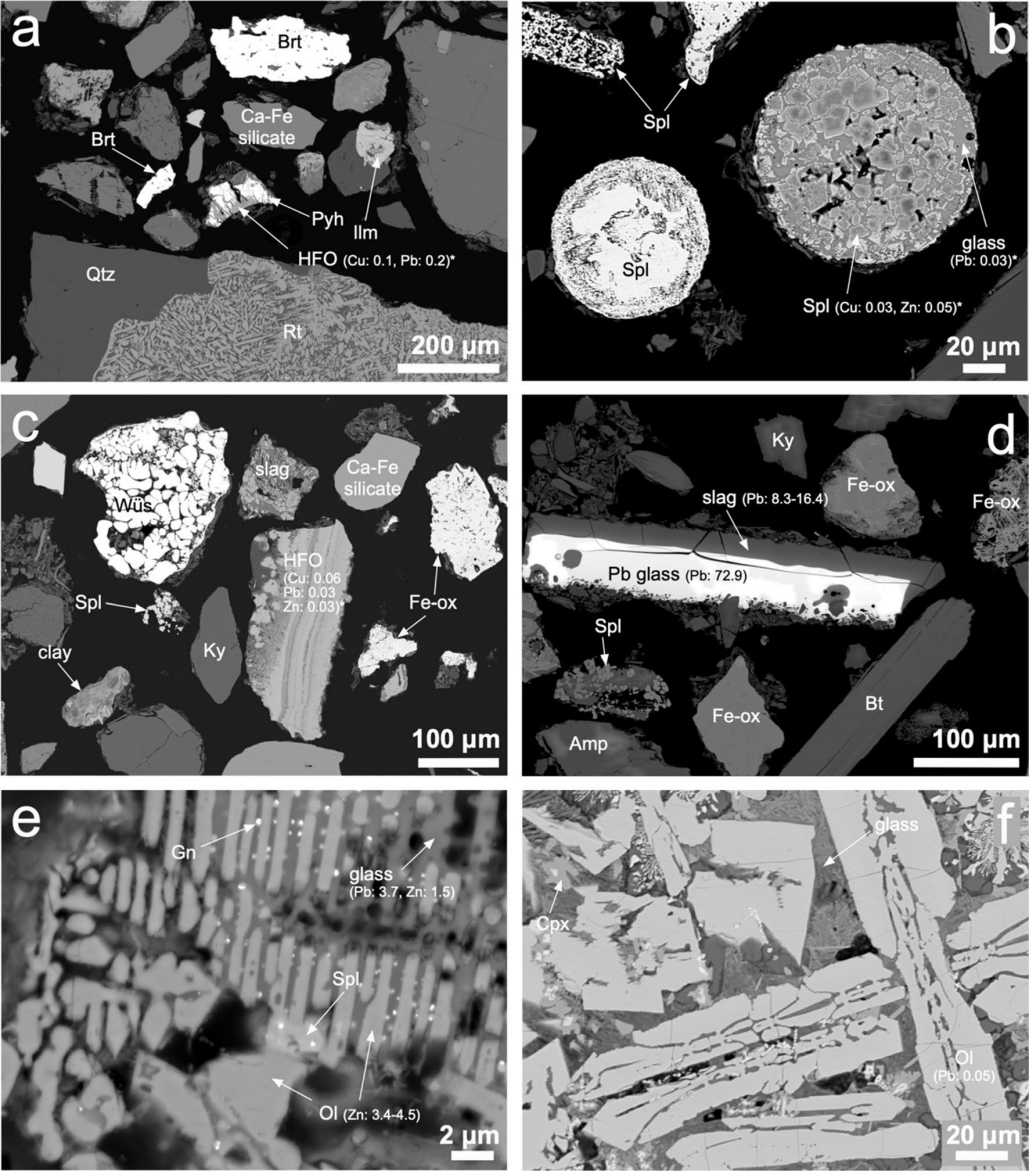
Scanning electron micrographs of the heavy mineral soil fractions in back-scattered electrons (a: sample J12; b-e: sample J15; f: sample J14). **a** Pyrrhotite (Pyh) grain weathered to metal-bearing hydrous ferric oxide (HFO) associated with heavy grains of barite (Brt) and rock fragments composed of rutile (Rt), quartz (Qtz), ilmenite (Ilm), and an unidentified Ca-Fe silicate. **b** Rounded particles derived from high-temperature processes (burning, smelting) composed of spinel (Spl) with a predominant magnetite composition (left) and slag-like particles consisting of metal-bearing spinel and glass. (**c**) Geogenic particles composed of silicates, such as clay minerals, kyanite (Ky), and unidentified Ca-Fe silicates in association with wüstite (Wüs), unidentified Fe oxides (Fe-ox), a slag fragment and metal-bearing secondary hydrous ferric oxides (HFO). **d** Fragment of Pb-rich glass surrounded by a Pb-bearing silicate slag and a weathered slag-like particle composed of spinel and glass in association with geogenic Fe oxides, kyanite, biotite (Bt) and amphibole (Amp). **e** Weathered slag particles composed of Zn-bearing olivine (Ol), spinel, and metal-bearing glass with entrapped submicrometric galena (Gn) inclusions. **f** Slag fragment with predominant skeletal crystals of olivine, clinopyroxene and a glassy matrix. Mineral abbreviations according to Warr (2021). Metal concentrations are indicated (in wt.% of oxides with *, in wt.% of metals with no asterisk)

Micro-XRF imaging was used to determine the metal(loid)-rich hotspots in the polished section prepared from the selected soil samples (Fig. S1). Despite some spectral overlaps (see the caption of Fig. S1), these locations were further examined by SEM/EDS and EPMA. Soil samples contained mineral fragments originating from the geological environment, such as quartz (SiO_2_), rutile (TiO_2_), barite (BaSO_4_), ilmenite (FeTiO_3_), kyanite (Al_2_SiO_5_), and other rock-derived silicates (including micas, clay minerals, and non-identified Ca-Fe alumosilicates of variable compositions). In addition to numerous bedrock minerals, pedogenic (e.g., secondary hydrous ferric oxides, HFOs) and anthropogenic particles (ashes, slag-like fragments) were observed (Fig. 3). Surprisingly, despite the location of the sampling spots near the ore veins mined in the history (e.g., samples J1 and J12), only a few sulfide grains were observed (Fig. 3a). Euhedral fragments of the pyrrhotite-like phase (Fe_1-x_S) contained small amounts of Cu (0.27 wt.%) and Pb (0.05 wt.%) (Fig. 3a; Table S5). Both metals were also detected in hydrous ferric oxides, forming a weathering rim on pyrrhotite grains (Fig. 3a; Table S5). Pedogenic metal-containing HFOs were relatively common phases in all the studied samples (Fig. 3c; Table S5). In contrast, other Fe oxides (e.g., wüstite, FeO, magnetite, Fe_3_O_4_, or non-identified Fe oxide minerals) were substantially less enriched in metals (e.g., Fig. 3c, d). HFOs that host Pb (and also other trace elements) are typically found in many soils in the vicinity of ore mining areas or other contaminated urban settings (Argyraki, 2014; Boisa et al., 2013; Drahota et al., 2018; Ettler et al., 2022; Hiller et al., 2024).

Spherical particles originating from high-temperature combustion (or metallurgical) processes were composed of spinel phases only and/or occurred as slag-like particles with spinel dendrites trapped within the metal-bearing silicate glass (Fig. 3b). Spinels are key host phases for Zn and Cr (Table S5). Slags of variable shapes and sizes documenting the pyrometallurgical history in Jihlava were also found in other samples – they either form a layer on the Pb glass fragments (Fig. 3d) or weathered particles with dendritic or skeletal crystals of olivine containing Zn, and Pb- and Zn-rich glass (Fig. 3e,f; Table S5). According to the EDS analyses, submicrometric heavy inclusions trapped within the slag glass corresponded to galena, PbS (Fig. 3e). We assume that the weathering features in the slag fragments, indicating their partial dissolution can result in the long-term release of contaminants into the soil environment (Fig. 3e,f). In contrast to Pb, Cr, Cu, and Zn, hosting phases for As, Cd, and V were not found, probably due to the substantially lower concentrations in the original soil samples.

### Bioaccessible concentrations and exposure estimates

The bioaccessible concentrations (mg/kg) and calculated bioaccessible fractions (%) are reported in Table S6. The bioaccessibilities and BAF values were very low for As (< 0.76–4.69 mg/kg, BAF up to 20%), Cd (0.15–1.75 mg/kg, the BAF could not be determined for any sample because the total concentrations were below the LOD), Cr (< 0.41–2.02, BAF up to 1%) and V (< 1.96–16.2 mg/kg, BAF up to 12%), indicating that these contaminants were tightly bound to the soil constituents and were not easily leachable in the SGF. The generally low leachabilities of Cr and V in the SGF agree well with other studies (Gbefa et al., 2011; Hiller et al., 2024; Kelepertzis et al., 2021; Reis et al., 2014b). We hypothesize that insoluble Cr-bearing spinels (Table S5) are responsible for the observed low Cr bioaccessibility. The As and Cd bioaccessibilities are similar to studies dealing with soil in standard urban settings (generally up to units of mg/kg; Gbefa et al., 2011; Hiller et al., 2024; Okorie et al., 2012) rather than more polluted sites, where these elements exhibit significantly higher bioaccessible concentrations and in some cases also BAF values (As: Drahota et al., 2018; Cd: Roussel et al., 2010 and Boisa et al., 2013).

On the contrary, Cu, Pb and Zn were substantially more bioaccessible; Cu in the range of 2.61–20.3 mg/kg (BAF 10–64% of the total, median: 25%), Pb in the range of 6.00–81.6 mg/kg (BAF 26–72%, median: 47%), and Zn in the range of 6.97–233 (BAF 4–56%, median: 22%). The bioaccessible concentrations and BAF values are in the ranges similar to those of other urban environment studies (Gbefa et al., 2011; Hiller et al., 2017, 2024; Okorie et al., 2012). Multiple phases hosted Cu, Pb, and Zn in our soil samples (Fig. 3; Table S5) and some of them could be the source of these metals during the extraction in the SGF.

While spinels (also important carriers of Zn) are expected to be relatively stable, the PHREEQC calculations indicated that Fe oxyhydroxides exhibit negative saturation indices (ferrihydrite, Fe(OH)_3_: -6.19 ± 0.25; goethite, FeOOH: -3.48 ± 0.25; n = 19) and tend to dissolve during the interaction of the soil with the SGF. Hiller et al. (2024) have also recently documented that during the bioaccessibility testing, Fe oxyhydroxides from urban soils covering a former municipal landfill could be readily attacked by the acidified glycine solution and are probably responsible for the release of the incorporated metals. In one of the samples, slag fragments composed of metal-containing silicates (olivine) and glass exhibit signs of weathering (Fig. 3) and could also be prone to dissolution in the SGF.

**Fig. 4:**
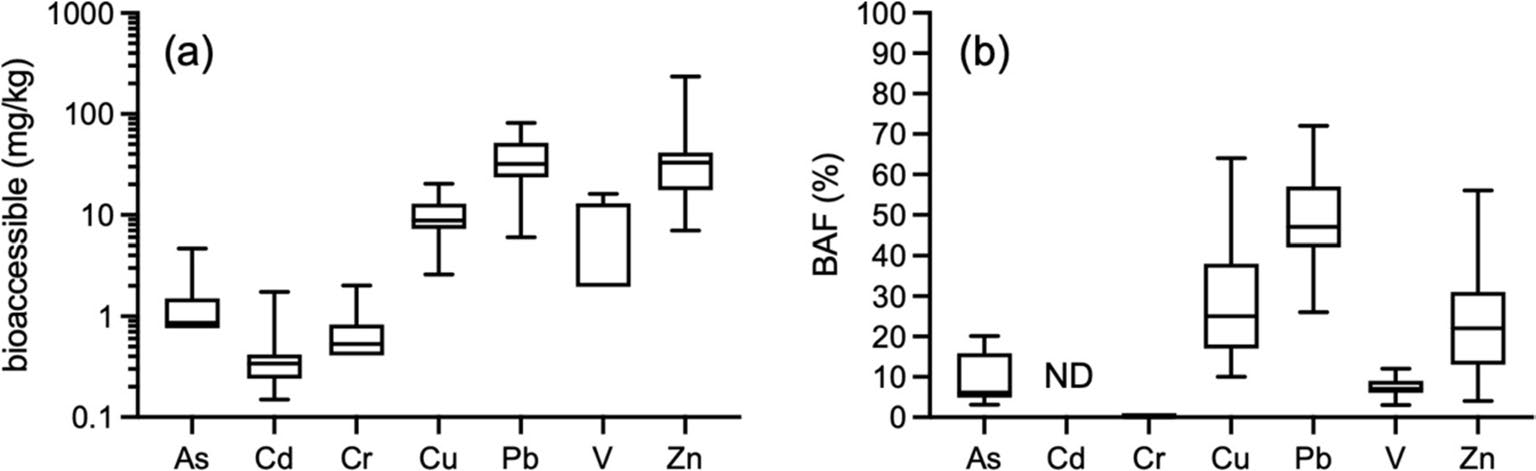
Box plots (min-Q1-median-Q3-max) of **a** the bioaccessible concentrations in mg/kg and **b** the bioaccessible fractions in % of the total concentrations for the metal(loid)s in the urban soils from the former mining town of Jihlava. Bioaccessible concentrations below the detection limits were set to the LOD values for plotting. ND = not determined (due to the total Cd concentration below the LOD)

**Fig. 5:**
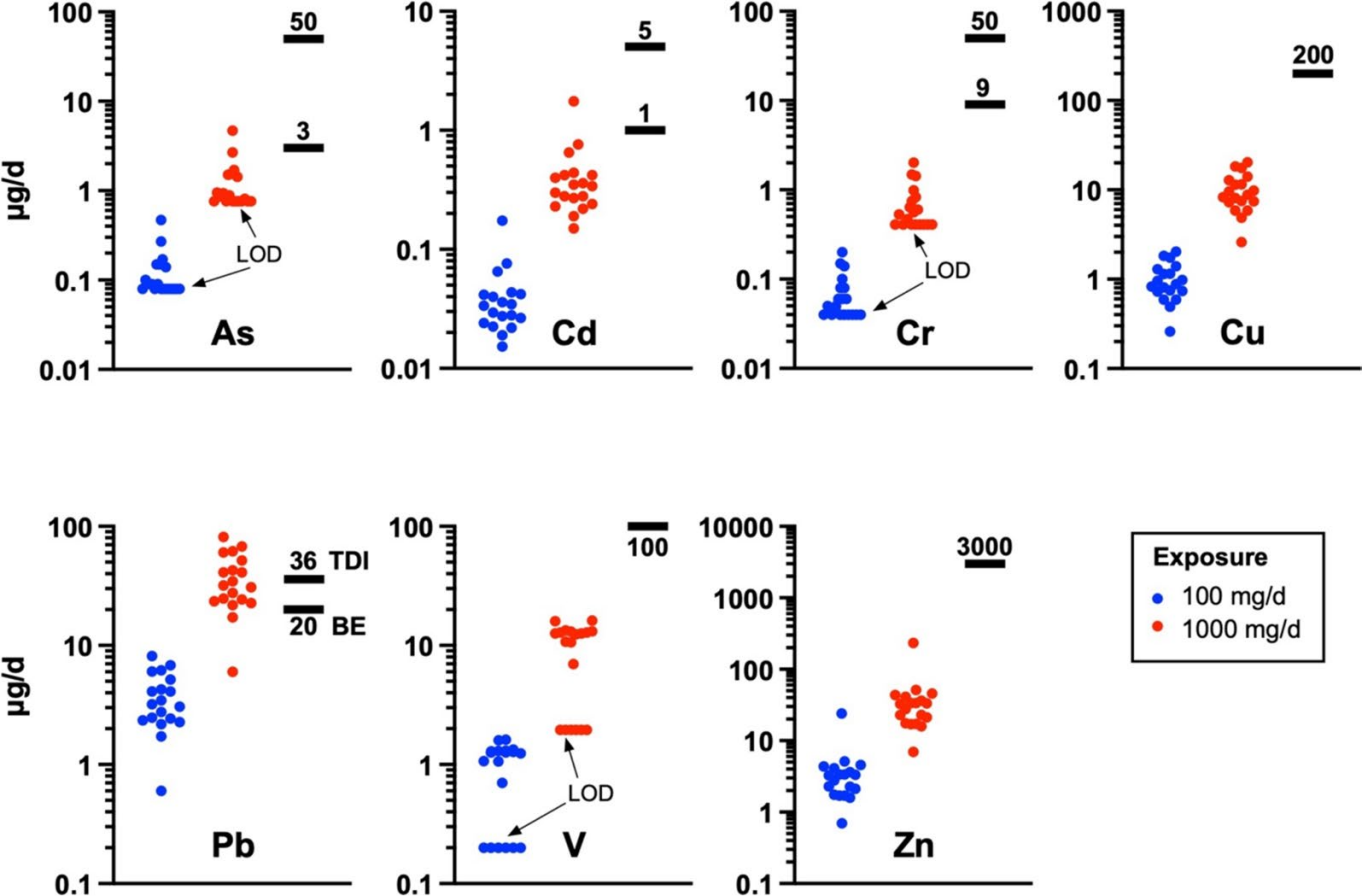
Exposure estimates calculated as daily intakes of individual contaminants (in ug/d) assuming a dust intake of 100 mg/d and 1000 mg/d and a comparison with the background exposure (BE), tolerable daily intake (TDI) limits (for Pb), and ATSDR minimal risk levels calculated for a 10-kg child (other contaminants). If not indicated, upper limit values correspond to “acute” exposure, and the lower limit values correspond to “chronic” or “intermediate” exposure. In the case of V, the limit value is for an “intermediate” exposure; for Cu and Zn, the “acute” and “chronic/intermediate” limits are identical (ATSDR, 2024)

When considering a 10-kg child as the target and a conservative soil ingestion rate (100 mg/d), the exposure calculations indicate that the contaminant intakes are far below the limits Fig. 4 defined by ATSDR (Fig. 5; Table S6).

For Pb, the daily intake is also far below the background exposure and TDI values defined by Baars et al. (2001) (Fig. 4; Table S6). Only when the ‘pica’ behavior is considered (we assume this is an unrealistic scenario for Central Europe), several samples exceed ATSDR’s minimal risk limits in the case of As and Cd (Fig. 4), and Pb could potentially be seen as being problematic, because 8 of 19 samples exceed the TDI limit of 36 µg/d.

### Environmental implications and future perspectives

Our data, based on a relatively small set of soil samples, provide the first insight into environmental risks in the residential areas of Jihlava, a town with a medieval mining history. While the total concentrations of contaminants are not excessively high, they increase significantly as particle size decreases (Fig. 2). It is also likely that the finest soil fractions (e.g., inhalable particulate matter, < 10 µm), which are particularly relevant for human health risk assessments (Menegaki et al., 2024), are even more enriched in inorganic contaminants (e.g., Drahota et al., 2018; Ettler et al., 2022). Although the oral bioaccessibility of metal(loid)s in the soil samples is relatively low, this study highlights the potential of the area for further environmental research. A "citizen science" approach, involving community-based sample collection, could be used to obtain more samples and cover a broader area. Furthermore, in addition to bioaccessibility testing and solid-speciation analysis used in this study, advanced techniques such as Pb isotopic tracing (Kelepertzis et al., 2021; Menegaki et al., 2024) and multivariate statistical analysis (Gabarrón et al., 2018; Parviainen et al., 2022) could be employed in future studies to better understand the sources and transport mechanisms of contaminants in this urban environment.

## Conclusions

The soils of the medieval silver mining town of Jihlava, the Czech Republic, collected near kindergartens, schools, playgrounds, and urban recreational areas, were studied to understand the effect of the old mining and ore processing activities on the distribution, binding, and oral bioaccessibility of metal(loid) contaminants. Despite the mining history in the Jihlava agglomeration, the total concentrations of metal(loid)s in the studied soils were not excessively high (up to 45.8 mg As/kg, 19.2 mg Cd/kg; 205 mg Cr/kg; 91.8 mg Cu/kg, 163 mg Pb/kg, 253 mg V/kg, 262 mg Zn/kg), although, in some cases, they exceeded the regulatory guidelines for agricultural and/or residential soils. The < 48-µm fraction used for the oral bioaccessibility testing exhibited significantly higher total metal(loid) concentrations than the original soils (< 2 mm). The SEM/EDS and EPMA investigations confirmed that the main hosts of Cu, Pb, and Zn in the soils were pedogenic Fe oxyhydroxides and slag-like particles, indicating that former metallurgical activities occurred in the area. These phases are especially expected to dissolve during the interaction with the simulated gastric fluid and are responsible for releasing bioaccessible metals. The exposure estimates for children (considered as the main targets) and assuming a soil ingestion rate of 100 mg/d confirmed that the daily intakes of metal(loid)s are far below the guidelines. Thus, the risk of human health risk based on incidental soil ingestion in the studied area seems quite limited. Future studies should involve a larger number of soil samples, for instance through a "citizen science" approach that engages the local community and covers a much broader area of this urban agglomeration. Additionally, more sensitive techniques, such as Pb isotopic measurements or advanced statistical methods, should be applied for a more detailed source apportionment of contaminants.

## Supplementary Information

Below is the link to the electronic supplementary material.

The MS Excel file contains information on sampling, QC/QA, EPMA analytical conditions and data, micro-XRF data, bulk concentrations and bioaccessibilities in soils, and exposure calculations.
Supplementary file1 (XLSX 732 KB)

## Data Availability

Datasets generated are available in the Supplementary Information file and are accessible in Zenodo data repository (10.5281/zenodo.14264861).

## References

[CR1] ATSDR (2024). Minimal Risk Levels (MRLs). Agency for Toxic Substances and Disease Registry, July 2024. Atlanta, USA. https://www.atsdr.cdc.gov/minimal-risk-levels/about/index.html

[CR3] Argyraki, A. (2014). Garden soil and house dust as exposure media for lead uptake in the mining village of Stratoni, Greece. *Environmental Geochemistry and Health,**36*, 677–692. 10.1007/s10653-013-9589-9 24292695 10.1007/s10653-013-9589-9

[CR4] Baars, A. J., Theelen, R.M.C., Janssen, P.J.C.M., Hesse, J.M., van Apeldoorn, M.E., Meijerink, M.C.M., Verdam, L., & Zeilmaker, M.J. (2001). *Re-evaluation of human-toxicological maximum permissible risk levels*. Bilthoven, the Netherlands: RIVM report 711701025.

[CR5] Bierkens, J., Van Holderbeke, M., Cornelis, C., & Torfs, R. (2011). Exposure Through Soil and Dust Ingestion. In F. A. Swartjes (Ed.), *Dealing with Contaminated Sites* (pp. 261–286). Springer Science+Business Media B.V.

[CR6] Boisa, N., Bird, G., Brewer, P. A., Dean, J. R., Entwistle, J. A., Kemp, S. J., & Macklin, M. G. (2013). Potentially harmful elements (PHEs) in scalp hair, soil and metallurgical wastes in Mitrovica, Kosovo: The role of oral bioaccessibility and mineralogy in human PHE exposure. *Environment International,**60*, 56–70. 10.1016/j.envint.2013.07.014 24013020 10.1016/j.envint.2013.07.014

[CR7] CCME (2024). *Canadian Environmental Quality Guidelines – Soil Quality Guidelines for the Protection of Environmental and Human Health*. Canadian Council of Ministers of the Environment. https://ccme.ca/en/summary-table

[CR8] Derner, K., Hrubý, P., Malina, O., & Večeřa, J. (2019). Hornické revíry vrcholného středověku a raného novověku ve srovnávacím pohledu. *Archaeologia Historica*. 10.5817/AH2019-2-18

[CR9] Dodd, M., Lee, D., Nelson, J., Verenitch, S., & Wilson, R. (2024). In vitro bioaccessibility round robin testing for arsenic and lead in standard reference materials and soil samples. *Integrated Environmental Assessment and Management, Advance Online Publication.*10.1002/ieam.4891 10.1002/ieam.4891 38329200

[CR10] Drahota, P., Raus, K., Rychlíková, E., & Rohovec, J. (2018). Bioaccessibility of As, Cu, Pb, and Zn in mine waste, urban soil, and road dust in the historical mining village of Kaňk, Czech Republic. *Environmental Geochemistry and Health,**40*, 1495–1512. 10.1007/s10653-017-9999-1 28620816 10.1007/s10653-017-9999-1

[CR2] EFSA (2009). Cadmium in food. Scientific opinion of the Panel on Contaminants in the Food Chain on a request from the European Commission on cadmium in food. *EFSA Journal,**980*, 1–139. 10.2903/j.efsa.2009.980

[CR45] EFSA (2010). Scientific opinion on lead in food. *EFSA Journal*, *8*(4), 1570. 10.2903/j.efsa.2010.1570

[CR11] Ettler, V., et al. (2022). Contaminant Binding and Bioaccessibility in the Dust From the Ni‐Cu Mining/Smelting District of Selebi‐Phikwe (Botswana). *GeoHealth*. 10.1029/2022GH000683 10.1029/2022GH000683 PMC9636585 36348990

[CR12] Ettler, V., Cihlová, M., Jarošíková, A., Mihaljevič, M., Drahota, P., Kříbek, B., Vaněk, A., Penížek, V., Sracek, O., Klementová, M., Engel, Z., Kamona, F., & Mapani, B. (2019). Oral bioaccessibility of metal(loid)s in dust materials from mining areas of northern Namibia. *Environment International,**124*, 205–215. 10.1016/j.envint.2018.12.027 30654327 10.1016/j.envint.2018.12.027

[CR13] Ettler, V., Kříbek, B., Majer, V., Knésl, I., & Mihaljevič, M. (2012). Differences in the bioaccessibility of metals/metalloids in soils from mining and smelting areas (Copperbelt, Zambia). *Journal of Geochemical Exploration,**113*, 68–75. 10.1016/j.gexplo.2011.08.001

[CR14] Ettler, V., Štěpánek, D., Mihaljevič, M., Drahota, P., Jedlicka, R., Kříbek, B., Vaněk, A., Penížek, V., Sracek, O., & Nyambe, I. (2020). Slag dusts from Kabwe (Zambia): Contaminant mineralogy and oral bioaccessibility. *Chemosphere,**260*, 127642. 10.1016/j.chemosphere.2020.127642 32683030 10.1016/j.chemosphere.2020.127642

[CR15] Fry, K. L., Wheeler, C. A., Gillings, M. M., Flegal, A. R., & Taylor, M. P. (2020). Anthropogenic contamination of residential environments from smelter As, Cu and Pb emissions: Implications for human health. *Environmental Pollution,**262*, 114235. 10.1016/j.envpol.2020.114235 32146362 10.1016/j.envpol.2020.114235

[CR16] GEMAS (2008). *EuroGeoSurveys Geochemical mapping of agricultural and grazing land soil of Europe (GEMAS) - Field manual*. Report 2008.038. Geological Survey of Norway, Trondheim, 46 p.

[CR17] Gabarrón, M., Faz, A., & Acosta, J. A. (2018). Use of multivariate and redundancy analysis to assess the behavior of metals and arsenic in urban soil and road dust affected by metallic mining as a base for risk assessment. *Journal of Environmental Management,**206*, 192–201. 10.1016/j.envman.2017.10.034 29065360 10.1016/j.jenvman.2017.10.034

[CR18] Galušková, I., Mihaljevič, M., Borůvka, L., Drábek, O., Frühauf, M., & Němeček, K. (2014). Lead isotope composition and risk elements distribution in urban soils of historically different cities Ostrava and Prague, the Czech Republic. *Journal of Geochemical Exploration,**147*, 215–221. 10.1016/j.gexplo.2014.02.022

[CR19] Gbefa, B. K., Entwistle, J. A., & Dean, J. R. (2011). Oral bioaccessibility of metals in an urban catchment, Newcastle upon Tyne. *Environmental Geochemistry and Health,**33*, 167–181. 10.1007/s10653-010-9330-x 20585835 10.1007/s10653-010-9330-x

[CR20] Goix, S., Uzu, G., Oliva, P., Barraza, F., Calas, A., Castet, S., Point, D., Masbou, J., Duprey, J. L., Huayta, C., Chincheros, J., & Gordon, J. (2016). Metal concentration and bioaccessibility in different particle sizes of dust and aerosols to refine metal exposure assessment. *Journal of Hazardous Materials,**317*, 552–562. 10.1016/j.jhazmat.2016.05.083 27344256 10.1016/j.jhazmat.2016.05.083

[CR21] Harvey, P. J., Taylor, M. P., Kristensen, L. J., Grant-Vest, S., Rouillon, M., Wu, L., & Handley, H. K. (2016). Evaluation and assessment of the efficacy of an abatement strategy in a former lead smelter community, Boolaroo, Australia. *Environmental Geochemistry and Health,**38*, 941–954. 10.1007/s10653-015-9779-8 26530186 10.1007/s10653-015-9779-8

[CR22] Hiller, E., Faragó, T., Kolesár, M., Filová, L., Mihaljevič, M., Jurkovič, L., Demko, R., Machlica, A., Štefánek, J., & Vítková, M. (2024). Metal(loid)s in urban soil from historical municipal solid waste landfill: Geochemistry, source apportionment, bioaccessibility testing and human health risks. *Chemosphere,**362*, 142677. 10.1016/j.chemosphere.2024.142677 38908448 10.1016/j.chemosphere.2024.142677

[CR23] Hiller, E., Mihaljevič, M., Filová, L., Lachká, L., Jurkovič, Ľ, Kulikova, T., Fajčíková, K., Šimurková, M., & Tatarková, V. (2017). Occurrence of selected trace metals and their oral bioaccessibility in urban soils of kindergartens and parks in Bratislava (Slovak Republic) as evaluated by simple in vitro digestion procedure. *Ecotoxicology and Environmental Safety,**144*, 611–621. 10.1016/j.ecoenv.2017.06.040 28645424 10.1016/j.ecoenv.2017.06.040

[CR24] Holub, M. (2015). Modelování historické primární produkce stříbra v hlavních rudních revírech Čech a přilehlé části Moravy (Results of the mathematic models of the historical silver production from main ore districts of the Bohemia and adjacent part of the Moravia). *Acta Rerum Naturalium,**18*, 9–20. (in Czech with English summary).

[CR25] Hrubý, P. (2019). *Metalurgická produkční sféra na Českomoravské vrchovině v závěru přemyslovské éry (Metallurgical production sphere in the Bohemian-Moravian Highlands at the end of the Přemyslid era)*. Opera Facultatis Philosophicae, no. 487, MUNI Press, Brno, 260 p. (in Czech with English summary)

[CR26] Hu, Y., Zhou, J., Du, B., Liu, H., Zhang, W., Liang, J., Zhang, W., You, L., & Zhou, J. (2019). Health risks to local residents from the exposure of heavy metals around the largest copper smelter in China. *Ecotoxicology and Environmental Safety,**171*, 329–336. 10.1016/j.ecoenv.2018.12.073 30616149 10.1016/j.ecoenv.2018.12.073

[CR27] Juhasz, A. L., Weber, J., & Smith, E. (2011). Impact of soil particle size and bioaccessibility on children and adult lead exposure in peri-urban contaminated soils. *Journal of Hazardous Materials,**186*, 1870–1879. 10.1016/j.hazmat.2010.12.095 21247691 10.1016/j.jhazmat.2010.12.095

[CR28] Kapusta, J., Dolníček, Z., Hrubý, P., & Malý, K. (2017). Strusky po tavbě polymetalických rud z locality Jihlava U Mlékárny (Slags after smelting of polymetallic ores from the locality Jihlava U Mlékárny). *Archeologia Technica,**28*, 28–32. (In Czech with English abstract).

[CR29] Kapusta, J., Dolníček, Z., Sracek, O., & Malý, K. (2022). Origin of historical Ba-rich slags related to Pb-Ag production from Jihlava Ore District (Czech Republic). *Minerals,**12*, 985. 10.3390/min12080985

[CR30] Kelepertzis, E., Chrastný, V., Botsou, F., Sigala, E., Kypritidou, Z., Komárek, M., Skordas, K., & Argyraki, A. (2021). Tracing the sources of bioaccessible metal(loid)s in urban environments: A multidisciplinary approach. *Science of the Total Environment,**771*, 144827. 10.1016/j.scitotenv.2020.144827 33529817 10.1016/j.scitotenv.2020.144827

[CR31] Koutek, J. (1952). O rudních žilách a starém dolování u Jihlavy (On the ore veins and the old mines at Jihlava). *Sborník ústředního geologického ústavu, oddíl geologický*, *19*, 77–116. (in Czech with English and Russian summary)

[CR32] Li, Y., Padoan, E., & Ajmone-Marsan, F. (2021). Soil particle size fraction and potentially toxic elements bioaccessibility: A review. *Ecotoxicology and Environmental Safety*, *209*, 111806. 10.1016/j.ecoenv.2020.111806 10.1016/j.ecoenv.2020.111806 33360288

[CR33] Madrid, F., Biasoli, M., & Ajmone-Marsan, F. (2008a). Availability and bioaccessibility of metals in fine particles of some urban soils. *Archives of Environmental Contamination and Toxicology,**55*, 21–32. 10.1007/s00244-007-9086-1 18058158 10.1007/s00244-007-9086-1

[CR34] Madrid, F., Díaz-Barrientos, E., & Madrid, L. (2008b). Availability and bio-accessibility of metals in clay fraction of urban soils of Sevilla. *Environmental Pollution,**156*, 605–610. 10.1016/j.envpol.2008.06.023 18653266 10.1016/j.envpol.2008.06.023

[CR35] Menegaki, S., Kelepertzis, E., Kypritidou, Z., Lampropoulou, A., Chrastný, V., Aidona, E., Bourliva, A., & Komárek, M. (2024). Characterization of the inhalable fraction (<10 µm) of soil from highly urbanized and industrial environments: Magnetic measurements, bioaccessibility, Pb isotopes and health risk assessment. *Environmental Geochemistry and Health,**46*, 320. 10.1007/s10653-024-02009-z 38849623 10.1007/s10653-024-02009-z PMC11161548

[CR36] Moya, J., & Phillips, L. (2014). A review of soil and dust ingestion studies for children. *Journal of Exposure and Environmental Epidemiology,**24*, 545–554. 10.1038/jes.2014.17 10.1038/jes.2014.17 24691008

[CR37] Okorie, A., Entwistle, J., & Dean, J. R. (2012). Estimation of daily intake of potentially toxic elements from urban street dust and the role of oral bioaccessibility testing. *Chemosphere,**86*, 460–467. 10.1016/j.chemosphere.2011.09.047 22024094 10.1016/j.chemosphere.2011.09.047

[CR38] Parkhurst, D.L., & Appelo, C.A.J. (2013). *Description of input and examples for PHREEQC version 3–A computer program for speciation, batch-reaction, one-dimensional transport, and inverse geochemical calculations.* U.S. Geological Survey Techniques and Methods, book 6, chap. A43.

[CR39] Parviainen, A., Vázquez-Arias, A., Arrebola, J. P., & Martín-Peinado, F. J. (2022). Human health risks associated with urban soils in mining areas. *Environmental Research,**206*, 112514. 10.1016/j.envres.2021.112514 34922981 10.1016/j.envres.2021.112514

[CR40] Pluskal, O., & Vosáhlo, J. (1998). Jihlavský rudní obvod (Jihlava mining district). *Vlastivědný sborník Vysočiny*, *13*, 157–191. (in Czech)

[CR42] Reis, A. P., Catinha, C., Noack, Y., Robert, S., & Dias, A. C. (2014b). Assessing human exposure to aluminium, chromium and vanadium through outdoor dust ingestion in the Bassin Minier de Provence, France. *Environmental Geochemistry and Health,**36*, 303–317. 10.1007/s10653-013-0964-5 23990126 10.1007/s10653-013-9564-5

[CR43] Reis, A. P. M., Cave, M., Sousa, A. J., Wragg, J., Rangel, M. J., Oliveira, A. R., Patinha, C., Rocha, F., Orsiere, T., & Noack, Y. (2018). Lead and zinc concentrations in household dust and toenails of the residents (Estarreja, Portugal): A source-pathway-fate model. *Environmental Science: Processes & Impacts,**20*, 1210–1224. 10.1039/c8em00211h 30084851 10.1039/c8em00211h

[CR41] Reis, A. P., Patinha, C., Noack, Y., Robert, S., Dias, A. C., & Ferreira da Silva, E. (2014a). Assessing the human health risk for aluminium, zinc and lead in outdoor dusts collected in recreational sites used by children at an industrial area in the western part of the Bassin Minier de Provence, France. *Journal of African Earth Sciences,**99*, 724–734. 10.1016/j.afrearsci.2013.08.001

[CR44] Roussel, H., Waterlot, C., Pelfrêne, A., Pruvot, C., Mazzuca, M., & Douay, F. (2010). Cd, Pb and Zn oral bioaccessibility of urban soils contaminated in the past by atmospheric emissions from two lead and zinc smelters. *Archives of Environmental Contamination and Toxicology,**58*, 945–954. 10.1007/s00244-009-9425-5 20016887 10.1007/s00244-009-9425-5

[CR46] Siciliano, S. D., James, K., Zhang, G., Schafer, A. N., & Peak, J. D. (2009). Adhesion and enrichment of metals on human hands from contaminated soil at an arctic urban brownfield. *Environmental Science and Technology,**43*, 6385–6390. 10.1021/es901090w 19746741 10.1021/es901090w

[CR47] Soto-Jiménez, M. F., Muñoz-Roos, S., Soto-Morales, S., Gómez-Lizarrága, L. E., & Bucio-Galindo, L. (2023). Environmental and health implications of Pb-bearing particles in settled urban dust from and arid city affected by Pb-Zn factory emissions. *Scientific Reports,**13*, 21287. 10.1038/s41598-023-48593-5 38042928 10.1038/s41598-023-48593-5 PMC10693616

[CR48] Tiesjema, B., & Baars, A.J. (2009). *Re-evaluation of some human-toxicological maximum permissible risk levels earlier evaluated in the period 1991–2001*. Bilthoven, the Netherlands: RIVM report 711701092.

[CR49] Tuhý, M., Hrstka, T., & Ettler, V. (2020). Automated mineralogy for quantification and partitioning of metal(loid)s in particulates from mining/smelting-polluted soils. *Environmental Pollution,**266*, 115118. 10.1016/j.envpol.2020.115118 32623271 10.1016/j.envpol.2020.115118

[CR50] Tyszka, R., Pedziwiatr, A., Pietranik, A., Kierczak, J., Ettler, V., Mihaljevič, M., & Zielinski, G. (2024). A long-term perspective on coal combustion solid waste interacting with urban soil. *Applied Geochemistry,**166*, 105975. 10.1016/j.apgeochem.2024.105975

[CR51] US EPA (2017). *SW-846 Test Method 1340. In Vitro Bioaccessibility Assay for Lead in Soil.* US EPA, Washington. https://www.epa.gov/hw-sw846/sw-846-test-method-1340-vitro-bioaccessibility-assay-lead-soil

[CR52] Vosáhlo, J. (1988). *Příspěvek k řešení strukturní pozice a minerogeneze hydrotermální polymetalické mineralizace na území rudních revírů Kamenná, Jihlava a Jezdovice. (Contribution to the understanding the structural position and minerogenesis of the hydrothermal polymetallic mineralization in ore districts Kamenná, Jihlava and Jezdovice).* MSc thesis, Charles University in Prague, Czech Republic, 191 p. + 15 maps (in Czech)

[CR53] Warr, L. N. (2021). IMA-CNMNC approved mineral symbols. *Mineralogical Magazine,**85*, 291–320. 10.1180/mgm.2021.43

[CR54] Yamamoto, N., Takahashi, Y., Yoshinaga, J., Tanaka, A., & Shibata, Y. (2006). Size distributions of soil particles adhered to children’s hands. *Archives of Environmental Contamination and Toxicology,**51*, 157–163. 10.1007/s00244-005-7012-y 16583253 10.1007/s00244-005-7012-y

[CR55] Özkaynak, H., Xue, J., Zartarian, V. G., Glen, G., & Smith, L. (2011). Modeled estimates of soil and dust ingestion rates for children. *Risk Analysis,**31*, 592–608. 10.1111/j.1539-6924.2010.01524.x 21039709 10.1111/j.1539-6924.2010.01524.x

